# Dectin-1-Independent Macrophage Phagocytosis of *Mycobacterium abscessus*

**DOI:** 10.3390/ijms241311062

**Published:** 2023-07-04

**Authors:** Alma E. Ochoa, Jack H. Congel, Jodi M. Corley, William J. Janssen, Jerry A. Nick, Kenneth C. Malcolm, Katherine B. Hisert

**Affiliations:** Department of Medicine, National Jewish Health, 1400 Jackson Street, Room A550, Denver, CO 80206, USA

**Keywords:** macrophage, Dectin-1, nontuberculous mycobacterium, phagocytosis, *Mycobacterium abscessus*, pattern recognition receptor

## Abstract

*Mycobacterium abscessus*, a species of nontuberculous mycobacteria (NTM), is an opportunistic pathogen that is readily cleared by healthy lungs but can cause pulmonary infections in people with chronic airway diseases. Although knowledge pertaining to molecular mechanisms of host defense against NTM is increasing, macrophage receptors that recognize *M. abscessus* remain poorly defined. Dectin-1, a C-type lectin receptor identified as a fungal receptor, has been shown to be a pathogen recognition receptor (PRR) for both *M. tuberculosis* and NTM. To better understand the role of Dectin-1 in host defense against *M. abscessus*, we tested whether blocking Dectin-1 impaired the uptake of *M. abscessus* by human macrophages, and we compared *M. abscessus* pulmonary infection in Dectin-1-deficient and wild-type mice. Blocking antibody for Dectin-1 did not reduce macrophage phagocytosis of *M. abscessus*, but did reduce the ingestion of the fungal antigen zymosan. Laminarin, a glucan that blocks Dectin-1 and other PRRs, caused decreased phagocytosis of both *M. abscessus* and zymosan. Dectin-1−/− mice exhibited no defects in the control of *M. abscessus* infection, and no differences were detected in immune cell populations between wild type and Dectin-1−/− mice. These data demonstrate that murine defense against *M. abscessus* pulmonary infection, as well as ingestion of *M. abscessus* by human macrophages, can occur independent of Dectin-1. Thus, additional PRR(s) recognized by laminarin participate in macrophage phagocytosis of *M. abscessus.*

## 1. Introduction

Nontuberculous mycobacteria (NTM) are considered opportunistic pathogens because they are ubiquitous in the environment [1] and do not cause disease in healthy individuals. However, NTM can cause infections in people with chronic airway diseases, and those with bronchiectasis have an up to 10,000-fold greater risk of infection compared to the general population [2]. Between 10 and 37% of people with chronic airways disease are estimated to have NTM chronically present in their airways, with *Mycobacterium avium* and *Mycobacterium abscessus* being the two predominant NTM species identified [3,4,5]. The basis for increased susceptibility to pulmonary NTM infections in people with airways diseases is likely multifactorial but remains poorly understood. This knowledge gap has become critical for several reasons. First, the prevalence of pulmonary NTM infections is increasing globally (a 2–6-fold increase between 1980s and 2010s) [3,6], with a marked increase in NTM in people with chronic airway diseases [4,5,7]. Second, pulmonary NTM infections worsen symptoms and outcomes in people with pre-existing lung diseases, including more rapid lung function decline and increased mortality [7,8]. Finally, pulmonary NTM infections, particularly those caused by *M. abscessus*, are difficult to treat, requiring administration of multiple antibiotics for months, with up to a 50% failure rate of eradication [4,9,10]. Accordingly, it is important to understand the mechanisms by which immune cells recognize and clear NTM from the lung.

Resistance to pulmonary NTM infections in the healthy lung relies on effective mucociliary clearance and intact innate immune responses, as defects in either increase susceptibility to these infections [11,12]. However, the ways in which immune cells in healthy lungs eliminate inhaled NTM remain a subject requiring continued study. Defense against mycobacteria is thought to be dependent on macrophages [13,14]. Considerable data exist regarding molecular mechanisms required to combat *Mycobacterium tuberculosis* infection [15,16,17]. It is unclear, though, if these findings can be extrapolated to NTM, as *M. tuberculosis* is an obligate intracellular pathogen, while NTM are opportunistic bacteria that can live outside the mammalian host. Several members of the C-type lectin receptor (CLR) family have been identified as important pattern recognition receptors (PRRs) used by macrophages to identify and mount inflammatory responses to mycobacteria; however, individual macrophage CLRs do not recognize all species of mycobacteria [17,18,19]. For example, Dectin-2 recognizes mannose-capped lipoarabinomannan (Man-LAM), a major lipoglycan found on the surface of *M. tuberculosis* and *M. intracellulare*, but does not recognize *M. abscessus* or *M. smegmatis*, neither of which employ mannose capping of lipoglycans [19]. Dectin-1, a CLR originally identified as an essential PRR for fungal antigens [20,21], has been shown to recognize several mycobacterial species including *M. tuberculosis*, *M. bovis*, *M. avium*, and *M. abscessus* [22,23,24], triggering macrophage intracellular signaling cascades and subsequent production of inflammatory cytokines [22,23,24]. One frequently cited study concluded that Dectin-1 is also essential for macrophage phagocytosis of *M. abscessus* [23]; however, macrophage PRRs that detect *M. abscessus* are overall less well studied than PRRs for *M. tuberculosis*. We sought to further characterize the role of Dectin-1 in host defense against initial infection with *M. abscessus* by evaluating the requirement for Dectin-1 during murine acute *M. abscessus* lung infection and human macrophage phagocytosis of *M. abscessus.*

## 2. Results

### 2.1. Blocking Antibody to Dectin-1 Reduces Phagocytosis of Zymosan by Human Monocyte Derived Macrophages but Does Not Decrease Uptake of M. abscessus

**Figure 1:**
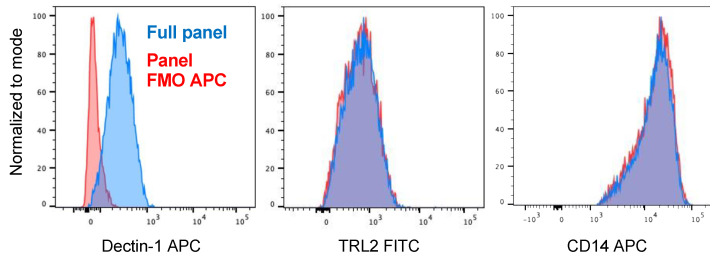
Dectin-1 is expressed by human monocyte-derived macrophages (MDMs). Human MDMs were stained for flow cytometry. Dectin-1+ cells were identified by comparing viable CD14+ cells stained with anti-Dectin-1 antibody (blue) or a fluorescence minus one (FMO) panel lacking anti-Decitn-1 antibody (red). TLR2 and CD14 are shown to demonstrate that detection of only Dectin-1 is altered in the FMO panel.

**Figure 2:**
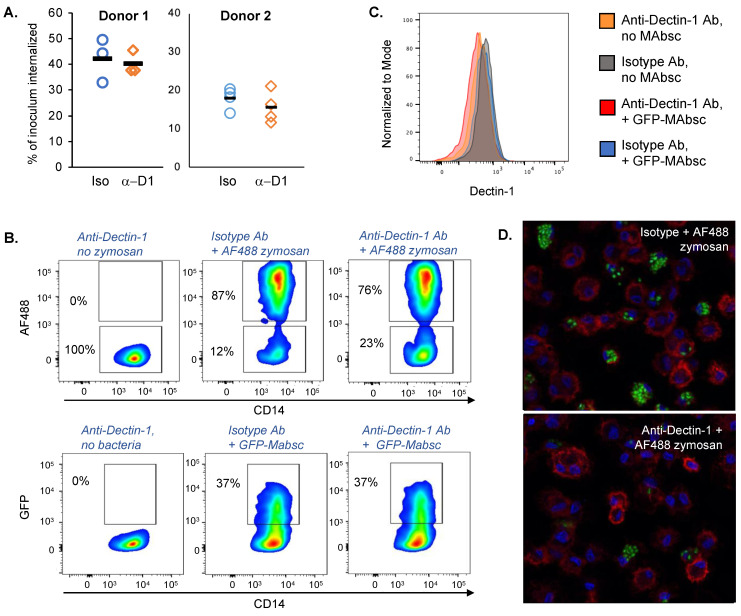
Blocking antibody for Dectin-1 does not impair MDM uptake of *M. abscessus*, but reduces phagocytosis of zymosan. MDMs were exposed to anti-Dectin-1 antibody (clone 259931) (α-D1) or isotype control (Iso) antibody, and then incubated with *M. abscesuss* or zymosan for 1 h. (**A**) Uptake of *M. abscessus* determined by quantification of intracellular colony forming units (CFUs). Each symbol represents a technical replicate. This experiment was repeated with MDMs from 4 different healthy donors, and representative results from 2 of the donors are shown. (**B**) Quantification of uptake of zymosan and *M. abscessus* determined by detection of fluorescent particles within macrophages by flow cytometry. Blue and green correspond to areas of lower cell density, red and orange are areas of high cell density, and yellow is mid-range. (**C**) Detection of Dectin-1 on the surface of MDMs by flow cytometry following treatment of MDMs with anti-Dectin-1 antibody (orange and red curve) or isotype control antibody (grey and blue curves). (**D**) Confocal fluorescent microscopy (63×) of representative images demonstrating decreased uptake of fluorescent zymosan particles in the presence of isotype control or anti-Dectin-1 blocking antibody. Blue = DAPI (stains DNA in nuclei); red = lectin (binds cell membranes); green = AF488 zymosan.

In people with chronic airways diseases and bronchiectasis, macrophages that accumulate in inflamed airways at sites of disease are primarily monocyte-derived macrophages (MDMs) recruited from the blood, rather than resident airspace or alveolar macrophages [25,26,27]. MDMs are therefore the macrophage population most likely to first interact with inhaled NTM. We thus chose to study the role of Dectin-1 on blood-derived human MDMs. Monocytes were isolated from healthy donors and matured to macrophages over seven days using macrophage-colony stimulating factor (M-CSF) as described [28]. Flow cytometry confirmed the expression of Dectin-1 on MDMs (Figure 1). To determine whether blocking Dectin-1 reduces phagocytosis of *M. abscessus*, we exposed MDMs to either a Dectin-1-specific blocking antibody (clone 259931) or isotype control antibody, infected cells with *M. abscessus* for 30 min, and then lysed the cells to determine internalized colony forming units (CFUs). As shown in Figure 2A, although there was variability in the uptake of *M. abscessus* by macrophages from different donors, there was no significant change in internalized *M. abscessus* when MDMs were pre-treated with Dectin-1 blocking antibody compared to the isotype control antibody.

To validate the ability of this specific antibody clone (259931) to block Dectin-1 [29,30], we evaluated whether it could reduce phagocytosis of zymosan, a β-glucan-rich cell wall component from the yeast *Saccharomyces cerevisiae* that requires Dectin-1 for ingestion [31]. We treated MDMs with either isotype control or clone 259931, and then incubated cells with green fluorescent protein (GFP)-expressing *M. abscessus* or fluorescently labeled (AF488) zymosan. Pre-treatment of MDMs with clone 259931 significantly reduced the uptake of AF488 zymosan by MDMs, but did not decrease the ingestion of GFP-*M. abscessus* (Figure 2B,D, Table 1). Furthermore, flow cytometry demonstrated a significant reduction in exposed Dectin-1 on the surface of MDMs treated with clone 259931 (with or without incubation with fluorescent microbial particles) compared to MDMs treated with the isotype control (Figure 2C, Table 1). These data demonstrate that the anti-Dectin-1 antibody clone 259931 blocks Dectin-1, obscuring it from subsequent antibodies that detect Dectin-1 and preventing Dectin-1 from binding the β-glucan-rich particle zymosan. Furthermore, human MDMs experience no impairment in the phagocytosis of *M. abscessus* following the specific blockade of Dectin-1.

### 2.2. Laminarin Decreases Phagocytosis of Both M. abscessus and Zymosan by Human Monocyte Derived Macrophages

**Figure 3:**
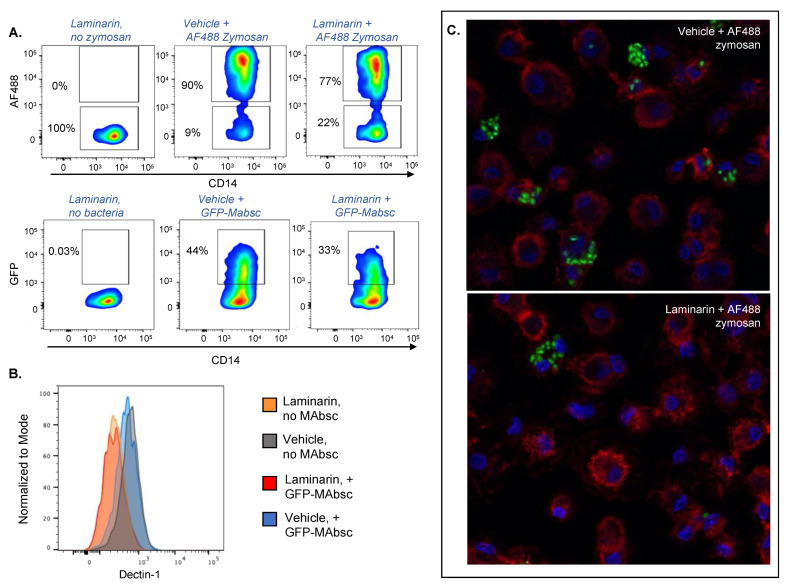
Laminarin reduces MDM uptake of both *M. abscessus and* zymosan. MDMs were exposed to laminarin or vehicle (DMSO), and then incubated with *M. abscesuss* or zymosan for 1 h. (**A**) Quantification of uptake of zymosan and *M. abscessus* determined by detection of fluorescent particles within macrophages by flow cytometry. Blue and green correspond to areas of lower cell density, red and orange are areas of high cell density, and yellow is mid-range. (**B**) Detection of Dectin-1 on the surface of MDMs by flow cytometry following treatment of MDMs with laminarin (orange and red curve) or vehicle (grey and blue curves). (**C**) Confocal fluorescent microscopy (63×) of representative images demonstrating decreased uptake of fluorescent zymosan particles in the presence of laminarin. Blue = DAPI (stains DNA in nuclei); red = lectin (binds cell membranes); green = AF488 zymosan.

In prior studies investigating the role of Dectin-1 in macrophage recognition of NTM, many authors have used the molecule laminarin to block Dectin-1 [22,23,24,32]. Laminarin is a low molecular weight β-glucan found in marine algae [33] which blocks Dectin-1 but has been shown to bind and block other PRRs and scavenger receptors as well [34,35]. Laminarin was found to decrease macrophage ingestion of *M. abscessus* [23]. Since we observed that specific blockade of Dectin-1 did not reduce phagocytosis of *M. abscessus* by MDMs, and prior studies investigating phagocytosis of *M. abscessus* used murine macrophages, we sought to determine whether laminarin decreases the ingestion of *M. abscessus* by human MDMs. We treated MDMs with laminarin or vehicle control (DMSO), and then incubated cells with GFP-expressing *M. abscessus* or AF488 zymosan. Laminarin reduced MDM phagocytosis of both zymosan particles and *M. abscessus* (Figure 3A,C, Table 2). As with the anti-Dectin-1 antibody clone 259931, the treatment of MDMs with laminarin decreased detectable Dectin-1 on the surface of MDMs (Figure 3B), confirming that laminarin blocks Dectin-1. These data, consistent with previously reported data from other researchers, suggest that a receptor (or receptors) that binds to and can be blocked by laminarin is required for macrophage phagocytosis of *M. abscessus*.

### 2.3. Dectin-1−/− Mice Do Not Exhibit Increased Susceptibility to M. abscessus Lung Infection

**Figure 4:**
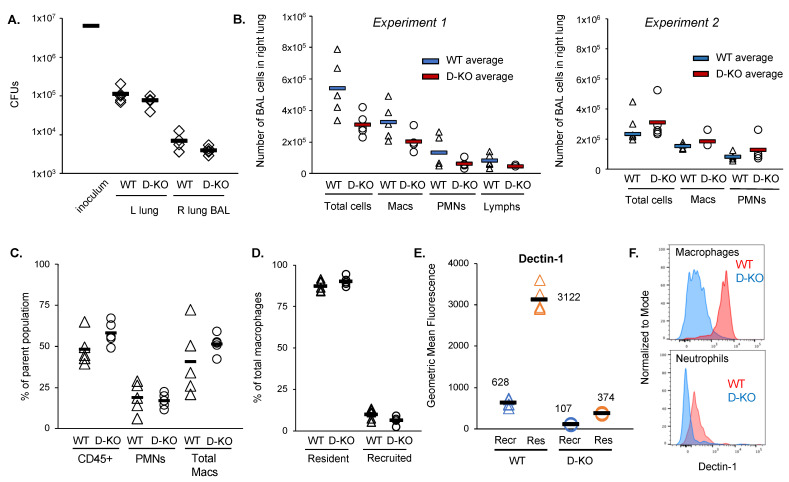
Dectin-1−/− mice do not exhibit increased susceptibility to pulmonary infection with *M. abscessus*. WT and Dectin-1−/− (D-KO) mice were infected via oropharyngeal inoculation with ~5 × 10^6^ CFU *M. abscessus*. Mice were sacrificed at day 7 after infection. (**A**) *M. abscessus* colony forming units (CFUs) were measured from bronchoalveolar lavage (BAL) fluid and lung homogenates. Black bars represent average values. (**B**) BAL cell numbers and differentials were quantitated by microscopy. More variability was detected in host cell numbers than in bacterial CFUs, so results from 2 different experiments are shown in panel (**B**). Triangles represent individual animal values for WT mice; circles represent individual animal values for Dectin−/− mice. BAL cells and lung homogenates were stained for flow cytometry to measure relative abundance of immune cell sub-populations in the airspaces in the lung parenchyma. (**C**) Relative abundance of total leukocytes (CD45+ cells), neutrophils (CD45+Ly6G+), and total macrophages (CD45+Ly6G−CD88+CD64+) in BAL. Triangles represent individual animal values for WT mice; circle represent individual animal values for Dectin−/− mice (**D**) Relative abundance of resident (CD11c+SigLecF+) and recruited (CD11c^−^SigLecF^−^) macrophages in BAL Triangles represent individual animal values for WT mice; circles represent individual animal values for Dectin−/− mice (**E**) Relative expression of Dectin-1 on the surface of resident and recruited macrophages as measured by geometric mean fluorescence index of PerCP-Cy5.5-Dectin-1 antibody. Triangles represent individual animal values for WT mice; circles represent individual animal values for Dectin−/− mice; blue indicates recruited (recr) macrophages, and orange indicates resident (res) macrophages. (**F**) Representative histograms demonstrating lack of Dectin-1 expression on macrophages (CD45+Ly6G−CD88+CD64+ cells) and neutrophils (CD45+Ly6G+ cells) isolated from Dectin-1 KO mouse BAL.

Our in vitro results using human macrophages demonstrate that Dectin-1 is not essential for the uptake *of M. abscessus*; however, work by others has shown that Dectin-1, in combination with TLR2, is required for macrophages to produce inflammatory cytokines in response to *M. abscessus* [22,23,24]. To determine whether Dectin-1 plays an essential role in host resistance to *M. abscessus* pulmonary infection, we infected wild-type and Dectin-1−/− mice with *M. abscessus* via the intratracheal route. Wild-type (WT) mice infected with between 10^6^ and 10^7^ colony forming units (CFUs) of *M. abscessus* cleared 1–2 logs of bacteria from the lungs during the first week of infection, with minimal CFUs remaining in the bronchoalveolar lavage (BAL) fluid at this time point (Figure 4A). Dectin-1−/− mice exhibited no differences in lung or BAL CFUs at one week after infection (Figure 4A). There were no differences detected between wild-type and Dectin-1−/− mice in total BAL cell numbers, or in relative or absolute abundance of BAL cells subsets as determined by microscopy (Figure 4B). Furthermore, staining of lung homogenates and BAL cells for flow cytometry revealed that the lack of Dectin-1 caused no alterations in proportions of immune cell populations in the lungs and in the airspaces following infection (Figure 4C,D, Table 3). The only difference detected between the WT and Dectin-1−/− mice was that Dectin-1 protein was not detectable on the surface of lung cells in the Dectin-1−/− mice (Figure 4E,F). These data indicate that Dectin-1 is not required for clearance of *M. abscessus* in a murine pulmonary infection model, and the absence of Dectin-1 does not appear to alter immune responses to *M. abscessus*.

## 3. Discussion

Inspired by prior studies describing Dectin-1 as an important PRR for *M. abscessus* [23,24], we evaluated the importance of Dectin-1 in recognition and phagocytosis of *M. abscessus* by macrophages and during a murine model of acute *M. abscessus* pulmonary infection. We found that human macrophages can ingest *M. abscessus* by methods independent of Dectin-1, and mice lacking Dectin-1 demonstrated no impairment in eradication of *M. abscessus* pulmonary infection. However, laminarin, a β-glucan that obstructs several PRRs in addition to Dectin-1, reduced macrophage phagocytosis of *M. abscessus*.

These data support two possible models of host recognition of *M. abscessus*. First, there may be redundancy in PRRs used by macrophages to phagocytose *M. abscessus*, and Dectin-1 is sufficient but not necessary to recognize and ingest *M. abscessus*. In support of this model, Shin et al. [23] found that over-expression of Dectin-1 in RAW264.7 cells resulted in enhanced uptake of *M. abscessus* compared to cells transfected with an empty vector. Alternately, a yet-to-be-identified receptor that is also blocked by laminarin may be the primary PRR used by macrophages to ingest *M. abscessus*, and this receptor may exhibit preferential binding to *M. abscessus* when multiple PRRs including Dectin-1 are present. Laminarin has been shown to block recognition of pathogens by receptors other than Dectin-1, including LYSMD3, CD11b, and ephrin-A2 (EphA2) [34,36,37,38], and thus one or more of these receptors may be the critical PRR for *M. abscessus*.

Dectin-1-deficient mice did not demonstrate increased susceptibility to acute pulmonary infection by *M. abscessus*, and no aberrant immune responses were detected in mice lacking Dectin-1. We acknowledge that our immune phenotyping did not include all immune pathways, and that Dectin-1−/− may manifest subtle differences in immune phenotypes after infection with *M. abscessus*. Furthermore, mice in which the *CLEC7A* gene (that encodes Dectin-1) has been disrupted in the germline, and have thus lacked Dectin-1 during development, may acquire compensatory upregulation of other receptors; however, if this compensation does occur, it would further support the theory that there is redundancy in PRRs that recognize *M. abscessus*, and that recognition can occur independent of Dectin-1. Our use of ex vivo receptor blocking experiments demonstrated that acute blockade of Dectin-1 did not impair macrophage phagocytosis, also consistent with Dectin-1-independent recognition mechanisms of *M. abscessus*.

Prior studies demonstrated that Dectin-1-deficient mice did not exhibit increased susceptibility to pulmonary *M. tuberculosis* infection [39]. Although several in vitro studies demonstrated that blocking Dectin-1 decreased macrophage and dendritic cell cytokine expression after exposure to *M. tuberculosis* [22,23,24], Dectin-1-deficient mice did not show increased bacterial burden in the lungs following aerosol infection with *M. tuberculosis*, and there were no observed changes in murine survival, histopathology of lung lesions, or inflammatory responses in *M. tuberculosis*-infected Dectin-1−/− mice compared to wild-type mice. In contrast, mice lacking the C-type lectin receptor CLEC4D (also known as MCL) were impaired in their control of bacterial replication with more CFUs in their lungs than WT mice after infection with either aerosolized H37Rv *M. tuberculosis* or *M. bovis* BCG, and pulmonary neutrophil infiltration increased [40]. Laminarin does not bind to or block CLEC4D, and thus CLEC4D is unlikely to be the PRR responsible for recognition and uptake of *M. abscessus*; however, these studies demonstrate the importance of extending in vitro host–pathogen interaction data to in vivo models of infection.

It should also be noted that cells may use distinct PPRs (or groups of PRRs), including Toll-like receptors (TLRs), C-type lectins, scavenger receptors, and others, to recognize different mycobacterial species [41,42,43,44]. As indicated above, although Dectin-1 was not essential for protection against murine infection with *M. tuberculosis* [17], CLEC4D/MCL was found to be important for murine defense against *M. tuberculosis* [40]. Dectin-2, which recognizes mannose-capped lipoarabinomannan (Man-LAM), participates in host recognition of *M tuberculosis* and *M. intracellulare*, but not *M. abscessus* or *M. smegmatis*, neither of which employ mannose capping of lipoglycans [19]. Although our data suggest that Dectin-1 may be dispensable for macrophage recognition of and host defense against *M. abscessus*, other work has shown a role for Dectin-1 in keratinocyte recognition and phagocytosis of *M. ulcerans* [32]. Finally, individual mycobacterial molecules may be recognized by several host PRRs or proteins: *M. avium* and some other species of NTM have been shown to produce fibronectin-binding proteins (FAPs) that interact with the host in multiple ways: adhering to extracellular matrix proteins [45], binding to integrins on host cells [46], and interacting directly with immune cell TLRs [47].

We acknowledge limitations to this study, which was focused on relatively acute events in host response to *M. abscessus*. It is possible that Dectin-1 could contribute to later stages of infection, or be relevant when different multiplicity of infection or greater inocula are used. Also, these studies were focused on the normal response to *M. abscessus*, relevant to the general population that is not susceptible to the infection. It is possible that in at-risk populations, Dectin-1-mediated responses to *M. abscessus* could be of greater (or less) clinical significance. We also note that we did not see complete inhibition of phagocytosis of *M. abscessus* by laminarin, or of zymosan by either laminarin or the anti-Dectin-1 antibody. The extent of blocking of PRRs with either laminarin or blocking antibodies is a stoichiometry challenge, and depends on both number of host cells and concentration of blocking reagent. Our studies showed a ~10% absolute reduction and a ~25% relative reduction in microbe or particle uptake when MDMs were incubated with laminarin at the concentrations we used. This is consistent with other studies in which laminarin was used to block the uptake of β-glucan particles and mycobacteria by different cell types (neutrophils, keratinocytes, and macrophages) [32,34,36].

In conclusion, our data demonstrate the existence of Dectin-1-independent pathways for macrophage recognition and phagocytosis of *M. abscessus*, an opportunistic pathogen with increasing global disease burden. These results highlight a fundamental gap in knowledge of how the healthy immune system eradicates *M. abscessus*, which is essential information for helping understand why specific populations, including those with chronic airways diseases, are at an increased risk for *M. abscessus* pulmonary infection.

## 4. Materials and Methods

### 4.1. Preparation of M. abscessus Inocula for Infections of Cells and Mice

*M. abscessus* ATCC 19977 smooth variant or *M. abscessus* ATCC 19977 smooth variant that expresses the green fluorescent protein (GFP) originally carried on the pBCM-2 vector were used to infect mice, and human-monocyte-derived macrophages (MDMs). Liquid aliquots of 7H9 Middlebrook media (Sigma-Aldrich, St. Louis, MI, USA, catalog number: M0178) supplemented with 10% of the Middlebrook ADC (albumin/dextrose/catalase) growth supplement (Millipore Sigma, Burlington, MA, USA, catalog number: M0553) were inoculated with *M. abscessus* or GFP-*M. abscessus*. Liquid cultures were incubated at 37 °C, shaking at 225 RPM over 5 days.

For infection of monocyte-derived macrophages (MDMs), stationary phase cultures (5-day liquid cultures) of *M. abscessus* or GFP-*M. abscessus* were sonicated 3–5 times for 1 s using a power of 3–5 (Fisher Scientific (Hampton, NH, USA) Sonic Dismembrator model 100). For inoculation of mice, *M. abscessus* stationary phase cultures were sub-cultured 1:5 in Middlebrook 7H9 broth base media supplemented with 10% Middlebrook ADC growth supplement, and grown for 17–20 h (on the day prior to infection). On the day of infection, sub-cultures were sonicated 3–5 times for 1 s using a power of 3–5. Following sonication, bacterial inocula for either MDMs or mice were pelleted 8000× *g* for 5 min, cells were washed 3 times with 1 mL of 1× PBS and resuspended in 500 μL of 1× PBS. Optical density was determined using a Beckman (Brea, CA, USA) DU 640 Spectrophotometer with absorbance set to 600 nm. *M. abscessus* cultures prepared as described above were diluted with a 1× PBS until an optical density (OD) of 1.0 was achieved. Prior experiments determined that an OD_600_ of 1.0 is equal to 2–4 × 10^8^ CFU/mL. Cultures were then diluted to achieve a final goal inoculum of a ~5 × 10^6^ CFU in 50 mL.

### 4.2. Preparation of Zymosan Inoculum

To prepare zymosan A (*S. cerevisiae*) BioParticles, Alexa Fluor 488 conjugate (Thermo Fisher (Waltham, MA, USA) catalog number: Z23373) lyophilized particles were reconstituted in 1 mL of 1× PBS, vigorously vortexed, and passed through a 27 G needle 5 times to disrupt clumps. Zymosan was then diluted in an RPMI 1640 medium + Glutamax supplemented with a 10% fetal bovine serum (FBS) and sonicated 3 times using a power of 3–5.

### 4.3. Isolation of Human Monocyte Derived Macrophages (MDMs)

Blood was obtained with IRB approval from healthy volunteers. PBMC were isolated from blood using density gradient centrifugation with Ficoll-Paque Plus (Cytiva; Marlborough, MA, USA, catalog number: 17144002). PBMCs were washed 3 times using cold 1× PBS with a 2.5 mM EDTA. Monocytes were isolated from PBMCs via negative selection using the pan monocyte isolation kit (Miltenyi Biotec; Gaithersburg, MD, USA, catalog number: 130-096-537) according to the manufacturer’s instructions using the manual isolation protocol. MDMs were plated at a density of 1.25 × 10^5^ cells/well in a 96-well plate or 2.5 × 10^5^ cells/well in a 48-well plate (for the quantitation of phagocytosis using bacterial CFUs) or 5 × 10^5^ cells in a 24-well plate for the quantitation of phagocytosis using flow cytometry. For confocal microscopy, 3 × 10^5^ monocytes were plated on glass coverslips. Monocytes were matured to MDMs as described [28]. Briefly, cells were cultured in an RPMI 1640 medium + Glutamax supplement (Fisher catalog number: 61-870-036) with 1% penicillin/streptomycin (Sigma catalog number: P4333) + 10% fetal bovine serum (FBS), and 100 ng/mL of recombinant human M-CSF (Peprotech; Rocky Hill, NJ, USA, catalog number: 30025) for 7 days. Media were replaced every two days.

### 4.4. Monocyte-Derived Macrophage Pathogen Recognition Receptor Blocking Assay

MDMs were washed 3× to remove residual penicillin/streptomycin, and then incubated for 1 h at 37 °C in a CO_2_ incubator with 5 μg/mL or 20 μg/mL of the Human anti-Dectin-1/CLEC7A Antibody (R&D Systems (R&D Systems (Minneapolis, MN, USA) catalog number: MAB1859, antibody clone #259931) or the Mouse IgG2B Isotype Control (R&D Systems catalog number: MAB004). Alternately, cells were incubated in laminarin 0.5–2 mg/mL (Thermo Fisher catalog number: J66193.MD) or 0.5–2% of DMSO as vehicle control for 1 h at 37 °C in a CO_2_ incubator. Cells were then washed, and media were added to MDMs containing either *M. abscessus* (MOI 10:1) or zymosan particles with a final inoculation ratio of ~0.5 (1.5 × 10^5^ particles added to 2.5 × 10^5^ MDMs). MDMs were centrifuged at 1000× *g* for 1 min to enhance microbial contact, then incubated for 30 min (or 60 min for MDMs on glass coverslips) at 37 °C in a CO_2_ incubator. After 30 min, media were changed on MDMs infected with *M. abscessus* to media containing amikacin 250 μg/mL to kill extracellular bacteria. For the uptake determined by intracellular CFUs, MDMs were washed 3 times with 1× PBS and lysed with 0.1% triton. Each sample was sonicated 3 times for 1 s (power of 3–5) before serial dilution and plating on LB agar. For flow cytometry, cells were washed with room temperature 1× PBS, and then detached by incubating in 4 °C 2.5 mM EDTA for 5 min followed by using a cell scraper, fixed blade (Cytoone, Ocala, FL, USA, catalog number: CC7600-0220).

### 4.5. Flow Cytometry of Monocyte-Derived Macrophages

Following detachment of MDMs from tissue culture plates, cells were incubated with FcR blocking reagent (Miltenyi Biotec catalog number: 130-059-901), and then stained using a mix of fluorochrome conjugated antibodies listed in Table 4. Cells were fixed with 4% PFA (Electron Microscopy Sciences; Hatfield, PA, USA, catalog number: 15710-S). Samples were acquired on an LSRII flow cytometer. Flow cytometry data were analyzed using the FlowJo v.10.7.1 Software. Gating strategy is shown in Supplementary Figure S1.

### 4.6. Staining of Monocyte-Derived Macrophages for Confocal Microscopy

After 1 h of incubation with GFP-*M. abscessus* or AF88-Zymosan, MDMs were washed 3 times. Lycopersicon Esculentum (Tomato) Lectin (LEL, TL), DyLight 594 (Thermofisher catalog number: L32471) was used to stain cell membranes. Cells were fixed with 4% PFA (Electron Microscopy Sciences catalog number: 15710-S). Coverslips were mounted Vectashield Antifade Mounting Medium with DAPI (Vector Laboratories (Newark, CA, USA) catalog number: H-1800). Slides were imaged using a Zeiss LSM 700 (Zeiss, Jena, Germany) confocal microscope.

### 4.7. Murine Model of M. abscessus Pulmonary Infection

All mice listed were approved by the Institutional Animal Care and Use Committee (IACUC) (Protocol number: AS2574-02-23) at National Jewish Health. The B6.129S6-Clec7atm1Gdb/J (Dectin-1 KO) and C57BL/6J (WT) mice strains were purchased from Jackson Labs. Male mice, 8–9 weeks of age, were inoculated with 50 μL containing 1 × 10^6^ to 5 × 10^6^ CFUs of *M. abscessus* via oropharyngeal aspiration. The murine pulmonary infection model was performed two times, with 5 mice per group in each experiment. Oropharyngeal inoculation was performed as described [48]. Briefly, mice were anesthetized mice with Fluriso isoflurane (VET one; Boise, ID, USA, catalog number: 501017) and positioned vertically. The tongue was gently pulled out of the mouth laterally to cover the esophagus and minimize obstruction of the trachea. *M. abscessus* in 50 μL PBS was deposited in the oropharynx; this inoculum was aspirated into the lungs during the mouse’s subsequent inhalation. On day 6 after infection, mice were euthanized via intraperitoneal injection of barbiturate solution (Fatal-Plus). The left lung main stem bronchus was tied off, and the left lung was removed for homogenization and plating for CFUs. Bronchoalveolar lavage (BAL) was performed on the right lung to plate for CFU, calculate total cell counts and differentials, and to use for flow cytometry. Following BAL, the right lung homogenate was perfused with saline to eliminate blood, recovered, and digested to make a single-cell suspension for flow cytometry.

### 4.8. Lung Tissue Homogenization to Determine Bacterial Burden

Lungs were bead beaten with 0.1% triton with stainless steel beads (Next Advance catalog number: SSB14B) using the bullet blender (Next Advance (Troy, NY, USA) catalog number: BT12LT) twice for 3 min at level 7–8. Homogenized lungs were serially diluted with 1× PBS and plated for CFUs on LB agar media plates (BD (Franklin Lakes, NJ, USA) catalog number: 244510), supplemented with 2.5 μg/mL of amphotericin B antifungal (Corning (Corning, NY, USA) catalog number: 30-003-CF).

### 4.9. Flow Cytometry on Murine Bronchoalveolar Lavage and Lung Tissue Homogenate Cells

The right lungs were minced into small pieces using scissors and incubated with 20% of liberase (Millipore Sigma catalog number: 05401127001) in HBSS for 25 min at 37 °C. All samples were kept on ice after this incubation. Lung digests were pressed through 70 μm cells to create single-cell suspensions. Cells were pelleted, resuspended, and cell numbers were quantitated. BAL and lung homogenate cells were resuspended with FcR block anti-mouse CD6/32 clone: 93 (Biolegend catalog number: 101320). Cells were then stained using a mix of fluorochrome conjugated antibodies shown in Table 5. Cells were washed and fixed with 4% PFA. Samples were acquired on an LSRII flow cytometer. Flow cytometry data were analyzed using the FlowJo v.10.7.1 Software (Ashland, OR, USA). Gating strategy is shown in Supplementary Data, Figures S2 and S3.

## Figures and Tables

**Table 1:** Effects of Dectin-1 blocking antibody on MDM ingestion of zymosan and *M. abscessus* and on levels of exposed Dectin-1 protein. Table shows mean values with (standard errors).

AF488 Zymosan			
Cell Type (% Parent Population)	Anti-Dectin-1 Antibody	Isotype Control Antibody	*p* Value
CD14+ cells	85.47 (1.6)	86.63 (0.9)	0.33
AF488+ CD14+ cells	76.17 (0.6)	86.43 (0.7)	4.3 × 10^−5^
AF488− CD14+ cells	23.03 (0.8)	12.63 (0.6)	5.9 × 10^−5^
**Geometric Mean Fluorescence Dectin-1**			
AF488+ CD14+ cells	203.33 (13.1)	244.67 (11.2)	0.014
AF488− CD14+ cells	319.33 (20.7)	444.67 (4.5)	0.00051
**GFP− *M. abscessus***			
**Cell Type** **(% parent population)**	**Anti-Dectin-1** **Antibody**	**Isotype Control** **Antibody**	***p* value**
CD14+ cells	88.00 (0.4)	87.87 (1.3)	0.87
AF488+ CD14+ cells	36.27 (2.8)	37.90 (2.6)	0.49
AF488− CD14+ cells	63.03 (2.9)	61.30 (2.2)	0.46
**Geometric Mean Fluorescence Dectin-1**			
AF488+ CD14+ cells	352.87 (16.0)	523.33 (8.0)	7.9 × 10^−5^
AF488− CD14+ cells	359.67 (9.3)	513.33 (16.8)	0.00016

**Table 2:** Effects of laminarin on MDM ingestion of zymosan and *M. abscessus* and on levels of exposed Dectin-1 protein. Table shows mean values with (standard errors).

AF488 Zymosan			
Cell Type (% Parent Population)	Anti-Dectin-1 Antibody	Isotype Control Antibody	*p* Value
CD14+ cells	84.50 (2.2)	87.87 (2.6)	0.15
AF488+ CD14+ cells	78.30 (1.9)	90.33 (0.3)	0.00040
AF488− CD14+ cells	21.13 (1.9)	9.12 (0.3)	0.00044
**Geometric Mean Fluorescence Dectin-1**			
AF488+ CD14+ cells	133.67 (12.0)	248.67 (4.9)	0.00011
AF488− CD14+ cells	231.67 (4.5)	443.33 (21.2)	7.1 × 10^−5^
**GFP− *M. abscessus***			
**Cell Type** **(% parent population)**	**Anti-Dectin-1** **Antibody**	**Isotype Control** **Antibody**	***p* value**
CD14+ cells	85.67 (1.0)	88.80 (0.90)	0.015
AF488+ CD14+ cells	34.30 (1.8)	42.70 (2.7)	0.011
AF488− CD14+ cells	64.57 (1.6)	56.37 (2.6)	0.0099
**Geometric Mean Fluorescence Dectin-1**			
AF488+ CD14+ cells	255.00 (3.0)	528.00 (19.5)	1.8 × 10^−5^
AF488− CD14+ cells	259.00 (2.7)	521.00 (9.6)	1.4 × 10^−6^

**Table 3:** Relative abundance of immune cell populations in the BAL and lungs of wild-type and Dectin-1−/− mice during acute pulmonary infection with *M. abscessus.* Table shows mean values with (standard errors).

Cell Type (% Parent Population)	Wild-Type Mice	Dectin-1−/− Mice	*p* Value
**BAL**			
CD45+	48.5 (1.0)	58.2 (7.1)	0.11
PMN	19.0 (9.3)	17.2 (4.3)	0.71
Total macrophages	40.9 (20.9)	51.5 (5.97)	0.29
% Dectin-1+ total macrophages	90.0 (3.5)	7.1 (1.4)	3 × 10^−11^
Resident macrophages	88.2 (3.8)	91.9 (2.5)	0.11
Recruited macrophages	10.5 (4.0)	7.0 (2.4)	0.12
**Lung Homogenate**			
CD45+	73.74 (6.8)	72.46 (4.8)	0.79
PMN	5.61 (1.9)	6.18 (0.8)	0.50
Total macrophages	10.53 (5.2)	11.64 (4.7)	0.75
Airspace macrophages	62.80 (12.4)	63.44 (9.8)	0.82
Non-airspace macrophages (recruited and interstitial)	35.30 (12.7)	34.72 (9.7)	0.84

**Table 4:** Flow cytometry antibodies used to stain human cells.

Fluorophore	Antibody Target	Clone	Company	Catalog Number
APC	CD14	61D3	Invitrogen (Waltham, MA, USA)	17-0149-42
PerCP Cy5.5	CD369 (Dectin-1)	15e2	Biolegend (San Diego, CA, USA)	355408
FITC	TLR2	TL2.2	Invitrogen	11.99.22.42
Calcein Blue-AM	Intact, viable cells	n/a	Invitrogen	65-0855 39

**Table 5:** Flow cytometry antibodies used to stain murine cells.

Fluorophore	Antibody Target	Clone	Company	Catalog Number
AlexaFluor 405	CD45	30.F11	Invitrogen	48045182
FITC	Ly-6G	1A8	BD Biosciences (Franklin Lakes, NJ, USA)	551460
PE	Siglec-F	E50-2440	BD Biosciences	552126
PE	CD88	20/70	Biolegend	135806
APC	CD88	20/70	Biolegend	135808
PerCP-eFluor 710	Clec7a/Dectin-1	bg1fpJ	Invitrogen	46-5859-82
PE-Cy7	CD64	X54-5/7.1	Biolegend	139314
APC-Cy7	CD11c	N418	Biolegend	117324
BUV395	Ly-6G	1A8	BD Biosciences	563978

## Data Availability

The data presented in this study are available on request from the corresponding author. Please send data requests to: Katherine Hisert, Department of Medicine, National Jewish Health; hisertk@njhealth.org.

## Supplementary Materials

The supporting information can be downloaded at: https://www.mdpi.com/article/10.3390/ijms241311062/s1.
