# Supplementary material for: Dectin-1-Independent Macrophage Phagocytosis of Mycobacterium abscessus
Source: Int J Mol Sci. 2023 Jul 4;24(13):11062. doi: 10.3390/ijms241311062 (PMC10341562; doi:10.3390/ijms241311062)

Supplementary Figure S1: flow cytometry analysis, gating strategy for human MDMs

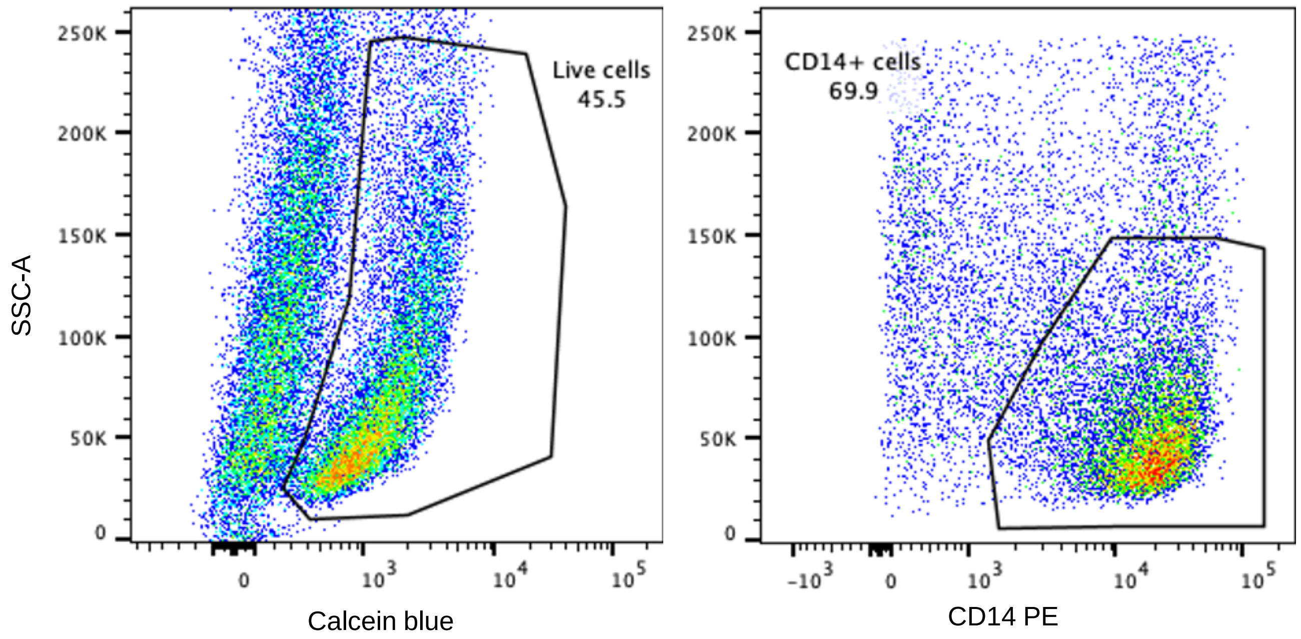

Supplementary Figure S2: flow cytometry analysis, gating strategy for mouse BAL

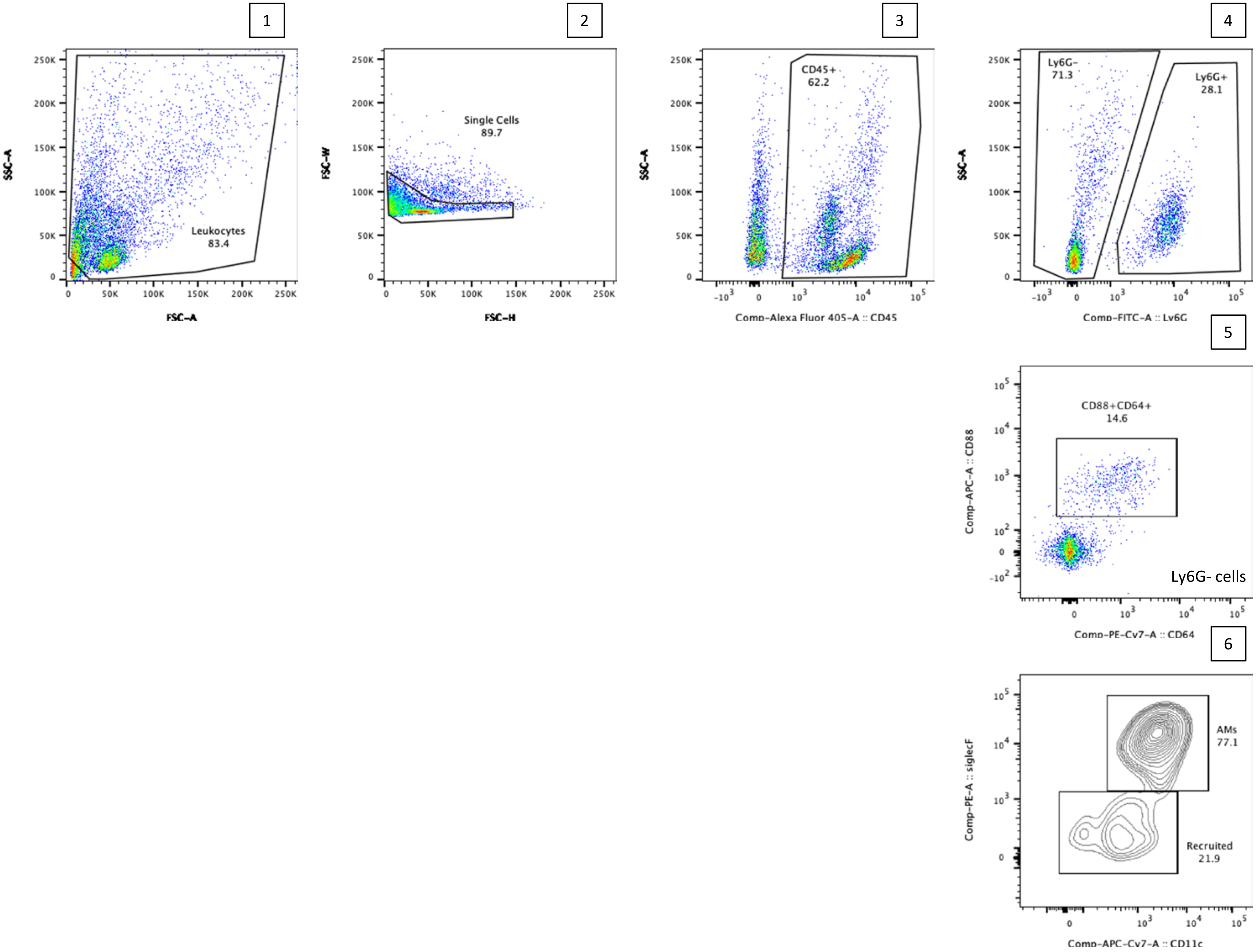

Supplementary Figure S3: flow cytometry analysis, gating strategy for mouse lung homogenates

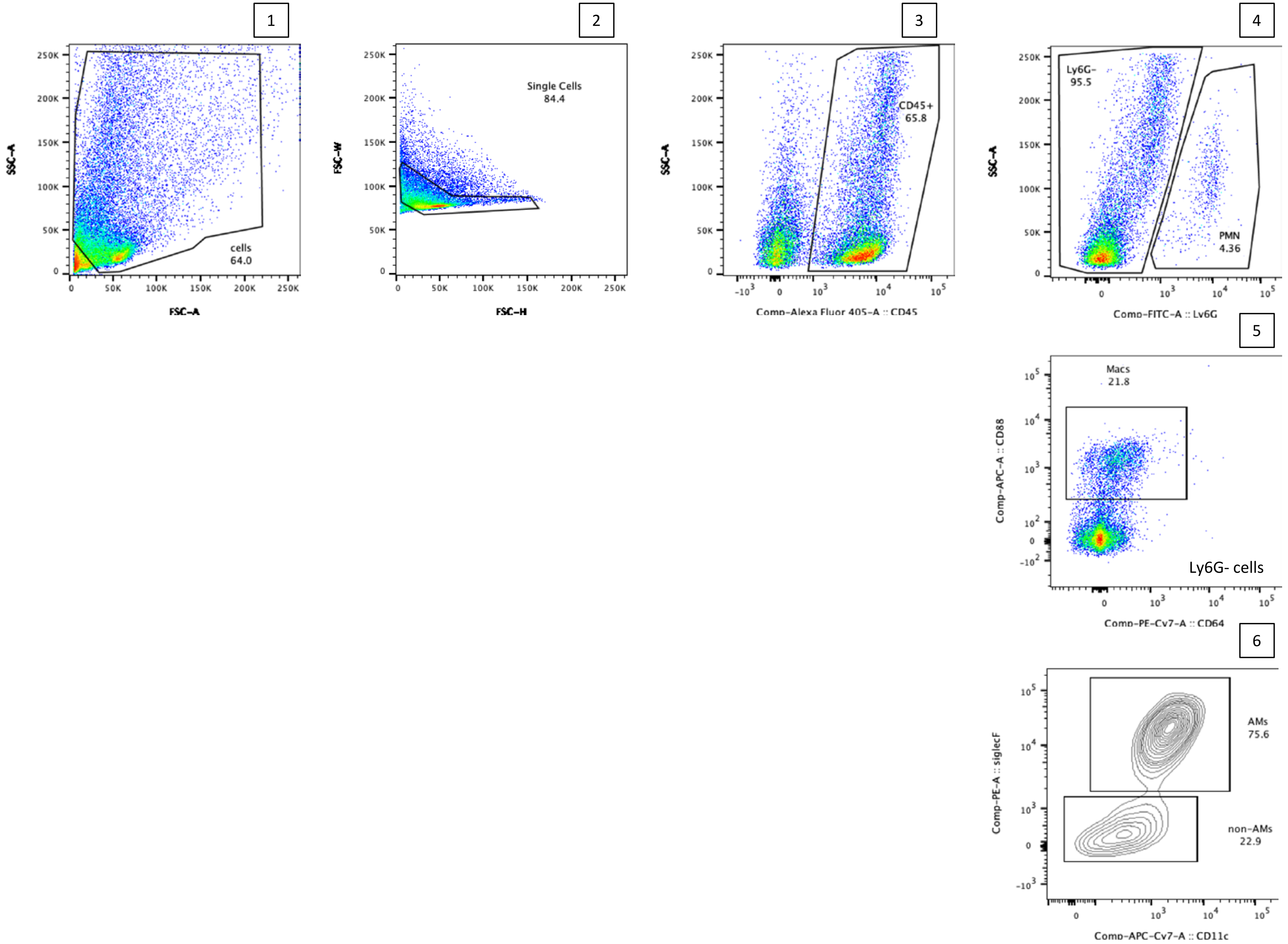

Supplement: Supplementary file 1 [file ijms-24-11062-s001.zip › ijms-2428513-supplementary.pdf]
